# Influence of Social Media Filters on Plastic Surgery: A Surgeon's Perspective on Evolving Patient Demands

**DOI:** 10.7759/cureus.80483

**Published:** 2025-03-12

**Authors:** Eduardo Miguel Veras, Samuel Román Ledesma, Joel Andres Acosta Matos, Manuel Emilio Castillo Cortorreal, Ilyana Goncharova, Rafael B. Rivera Bonilla, Andreina Rosario Rosario, Manuel De Jesus Encarnación Ramirez

**Affiliations:** 1 Plastic Surgery, Centro Medico Monumental, Santo Domingo, DOM; 2 Plastic Surgery, Dr. Ramon de Lara Military University Hospital, Santo Domingo, DOM; 3 Surgery, Hospital Casa do Portugal, Río de Janeiro, BRA; 4 Plastic Surgery, Dr. Oswaldo de Castro, São Paulo, BRA; 5 Plastic Surgery, Heartman Clinic, Moscow, RUS; 6 Plastic Surgery, Centro de Cirugía Plástica y Lipoescultura (CECILIP), Santo Domingo, DOM; 7 Medical School, Autonomous University of Santo Domingo, Santo Domingo, DOM; 8 Neurosurgery, Russian People's Friendship University, Moscow, RUS; 9 Human Anatomy and Histology, N.V. Sklifosovskiy Institute of Clinical Medicine, Moscow, RUS

**Keywords:** body dysphoria, lips surgery, plastic surgery, positive social media, social media

## Abstract

Introduction: Social media filters have significantly influenced contemporary beauty standards, leading to a surge in patients seeking plastic surgery to emulate these digitally altered appearances. Platforms like Instagram and TikTok are primary drivers of this trend, creating unrealistic expectations among patients who desire outcomes that mimic their filtered images.

Material and Methods: A survey conducted from June to August 2024 via Google Forms targeted plastic surgeons across various regions. The survey aimed to understand how social media filters impact patient requests and surgeon practices, collecting data on demographics, professional backgrounds, and patient outcomes.

Results: The survey, with 68 responses, revealed that social media filters are frequently referenced during consultations, influencing patient expectations and increasing the prevalence of revision surgeries. Surgeons reported significant ethical concerns and noted higher rates of psychological conditions such as body dysmorphic disorder among these patients.

Conclusion: Social media filters have reshaped patient demands in plastic surgery, necessitating ethical considerations and professional adaptations. Surgeons must balance patient desires with realistic outcomes, emphasizing education and psychological screening to address the challenges posed by this trend.

## Introduction

In recent years, the advent of social media has brought about significant changes in various aspects of society, with one of the most notable impacts being on perceptions of beauty and self-image. The widespread use of social media filters--tools that allow users to digitally alter their appearance in photos and videos--has reshaped the beauty landscape, fostering new standards that are often unattainable without cosmetic intervention. This phenomenon has given rise to what some have termed “Snapchat dysmorphia,” a condition where individuals seek plastic surgery to emulate the digitally enhanced versions of themselves seen through filters [1]. As a result, plastic surgeons are increasingly encountering patients whose expectations are heavily influenced by these altered images. This shift presents both opportunities and challenges for the field of plastic surgery, as surgeons must navigate the evolving demands of patients while maintaining ethical standards and ensuring realistic outcomes [2].

The rise of social media platforms like Instagram, TikTok, and Snapchat has been instrumental in popularizing these filters, which can instantly modify facial features, smooth skin, and even change the shape of one’s face. While these tools can be fun and harmless for casual use, they also have a profound impact on how individuals perceive themselves and others. Studies have shown that exposure to idealized images on social media can lead to increased body dissatisfaction and a desire to conform to these unrealistic standards [1,2]. This has led to a surge in the number of individuals seeking cosmetic procedures to replicate the filtered versions of themselves, often driven by the desire to achieve a more “Instagrammable” appearance [2].

Plastic surgeons have observed a marked increase in the number of patients referencing social media filters during consultations [3]. The most commonly referenced platforms include Instagram, TikTok, and Snapchat, where users are frequently exposed to images of influencers and celebrities who have undergone digital alterations. These platforms have become the primary venues for the dissemination of contemporary beauty standards, often characterized by features such as high cheekbones, full lips, and smooth, poreless skin [4]. The desire to emulate these features has driven many individuals to seek cosmetic procedures, leading to a growing trend of “social media-inspired” plastic surgery.

One of the key challenges plastic surgeons face in this new landscape is managing patient expectations. Social media filters can create a distorted sense of reality, where the line between what is digitally possible and what can be achieved through surgery becomes blurred. This has led to an increase in unrealistic expectations among patients, who may believe that surgery can provide results identical to those seen on their screens. Surgeons must therefore take on the dual role of clinician and educator, helping patients understand the limitations of surgery and guiding them toward realistic goals [5]. This process often involves using tools such as digital morphing software to create a visual representation of potential outcomes, which can help bridge the gap between patient desires and achievable results [6].

The ethical implications of social media filters in the context of plastic surgery are profound. The pursuit of a “filtered” appearance raises concerns about the promotion of homogenous beauty standards and the potential psychological impact on individuals who may already be vulnerable to body image issues. Body dysmorphic disorder (BDD), a condition characterized by an obsessive focus on perceived flaws in one’s appearance, is particularly prevalent among patients seeking cosmetic procedures influenced by social media. Studies have found that up to 15% of individuals seeking plastic surgery meet the diagnostic criteria for BDD, a rate significantly higher than in the general population [7]. This underscores the importance of psychological screening and assessment as part of the pre-surgical consultation process, ensuring that patients are making informed decisions based on realistic expectations and sound mental health [8].

The influence of social media filters also raises important questions about the role of plastic surgeons in shaping contemporary beauty standards. As gatekeepers of aesthetic procedures, surgeons have a responsibility to promote diversity and individuality in their practice, rather than reinforcing narrow ideals of beauty. This requires a nuanced understanding of the cultural context in which these demands are made, as well as a commitment to ethical practice. Surgeons must balance the desire to satisfy patient requests with the need to provide care that is in the patient’s best interest, both physically and psychologically [9]. In some cases, this may involve refusing a procedure if it is deemed likely to exacerbate existing mental health issues or if the patient’s expectations are deemed unrealistic.

The growing influence of social media on plastic surgery also highlights the need for increased regulation and oversight. Many plastic surgeons advocate for stricter guidelines governing the use of filters in social media advertising, as well as greater transparency in the portrayal of cosmetic procedures online. This could help mitigate the impact of these platforms on body image and reduce the pressure individuals feel to conform to unrealistic standards [10]. Additionally, there is a strong case for the development of ethical guidelines within the plastic surgery community to address the challenges posed by social media influences. These guidelines could provide a framework for managing consultations, using digital tools, and addressing the psychological aspects of patient care [11,12].

## Materials and methods

This study was conducted through a survey distributed via Google Forms from June 2024 to August 2024. The survey targeted plastic surgeons practicing across various regions: North America (United States, Canada), Europe (United Kingdom, Germany, France, Spain), Africa (Egypt), Asia (India), and Latin America (Mexico, Brazil, Argentina, Venezuela, Colombia, and the Dominican Republic), with the aim of gathering data on the influence of social media filters on patient requests and expectations. The survey was designed to capture a broad range of perspectives, including those of surgeons at different stages of their careers and with varying areas of specialization.

Participant selection and inclusion and exclusion criteria

The study included board-certified plastic surgeons specializing in cosmetic surgery. The qualification criteria required surgeons to have at least five years of independent clinical experience in aesthetic procedures; active involvement in social media-based patient consultations or marketing; experience with patient cases where social media filters influenced aesthetic requests

Exclusion criteria included surgeons without social media exposure in their practice, those primarily performing reconstructive (non-cosmetic) surgeries, and those without direct patient consultation experience.

Survey design and distribution

The survey was developed by a panel of experts in plastic surgery and medical ethics to ensure relevance and clarity. Prior to distribution, the survey underwent a pilot test with four board-certified plastic surgeons who provided feedback on ambiguities, question structure, and overall usability. Based on their input, minor revisions were made to enhance clarity and ensure that the survey accurately captured the intended data.

To validate the survey, content validity was assessed by an independent group of two plastic surgeons, who reviewed the questions for relevance, clarity, and alignment with the study objectives. Their feedback led to refinements in wording and response options.

Survey structure and question types

The final survey included a mix of question types to capture both quantitative and qualitative insights: Likert-scale questions (e.g., assessing the perceived impact of social media filters on patient expectations); multiple-choice questions (e.g., identifying common types of social media-altered images referenced by patients); open-ended questions (e.g., allowing surgeons to describe ethical dilemmas and challenges in consultations)

Data collection and analysis

A total of 116 surveys were distributed via email to plastic surgeons in various regions (America, Asia, Europe, Africa, and Australia), with follow-up reminders sent to encourage participation. Responses were collected anonymously to ensure that participants felt comfortable providing honest and accurate information. A total of 68 responses were received, yielding a response rate of 58.6%, and included in the final analysis.

Data from the survey was analyzed using descriptive statistics, with the results presented in terms of percentages and frequencies. Cross-tabulation was used to explore relationships between different variables, such as the correlation between years in practice and the frequency of social media filter references during consultations. The data was then synthesized to identify key trends and patterns, which were used to inform the discussion and conclusions of the study.

Anonymity and data confidentiality

To protect participant confidentiality, the survey was conducted anonymously, and no identifying information, IP addresses, or institutional affiliations were collected. Participants were explicitly informed that their responses would remain confidential and be used solely for research purposes, mitigating concerns of professional stigma or ethical scrutiny.

Ethical considerations

This study was conducted in accordance with ethical guidelines for survey research, including obtaining informed consent from all participants. Participants were informed of the purpose of the survey and assured that their responses would be kept confidential.

## Results

A total of 116 surveys were distributed, with 68 completed responses, yielding a response rate of 58.6%. Participants were board-certified plastic surgeons actively practicing in aesthetic surgery. The findings are organized into several key areas: demographic and professional background, the influence of social media on patient expectations, ethical considerations, psychological impacts, professional practices and adaptations, and patient outcomes.

Demographic and professional background

**Figure 1:**
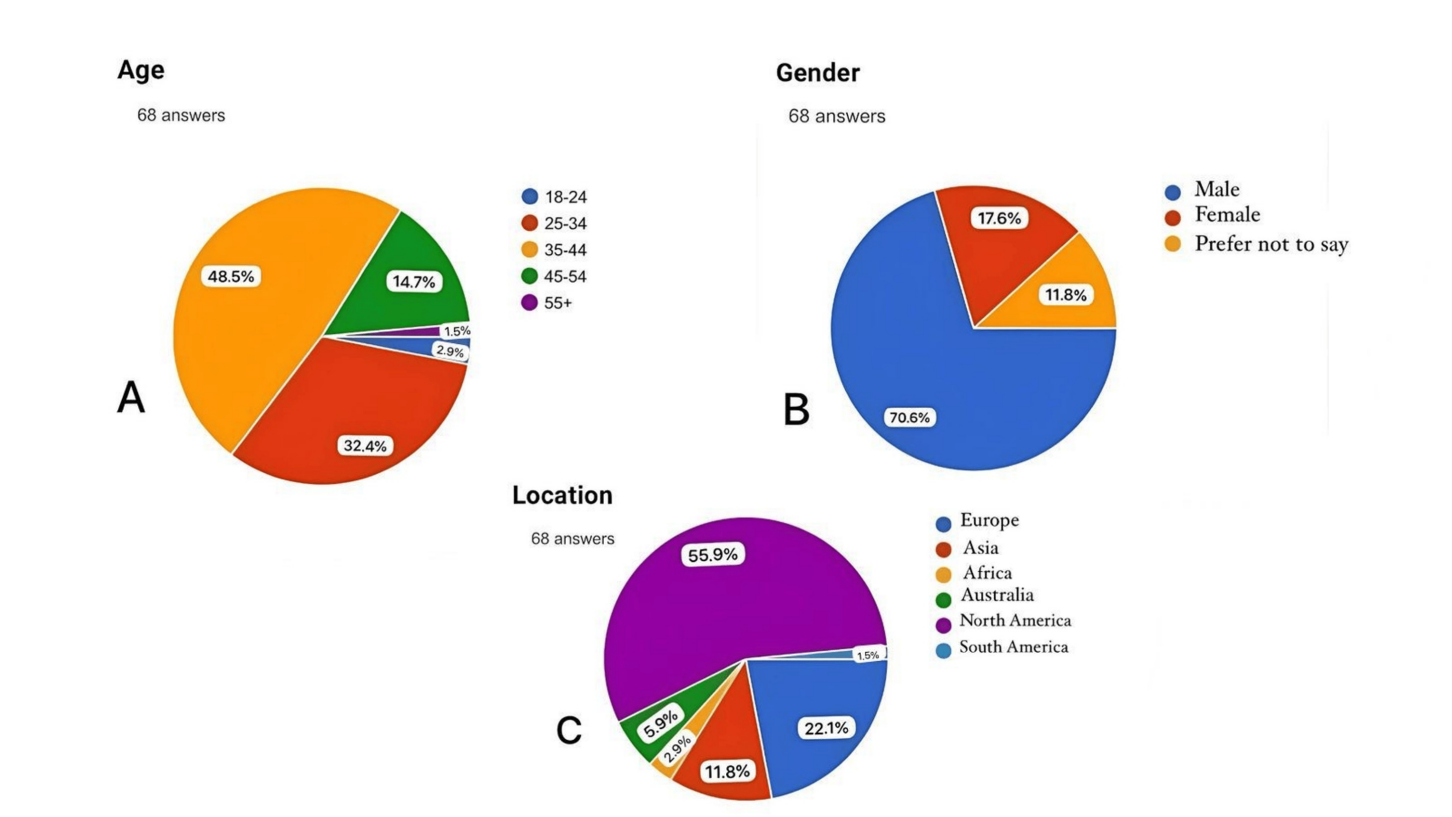
Demographic Background of participants. (A) age; (B) gender; (C) location NOTE: Since only respondents from the United States participated, 'North America' in the figure refers exclusively to them.

**Figure 2:**
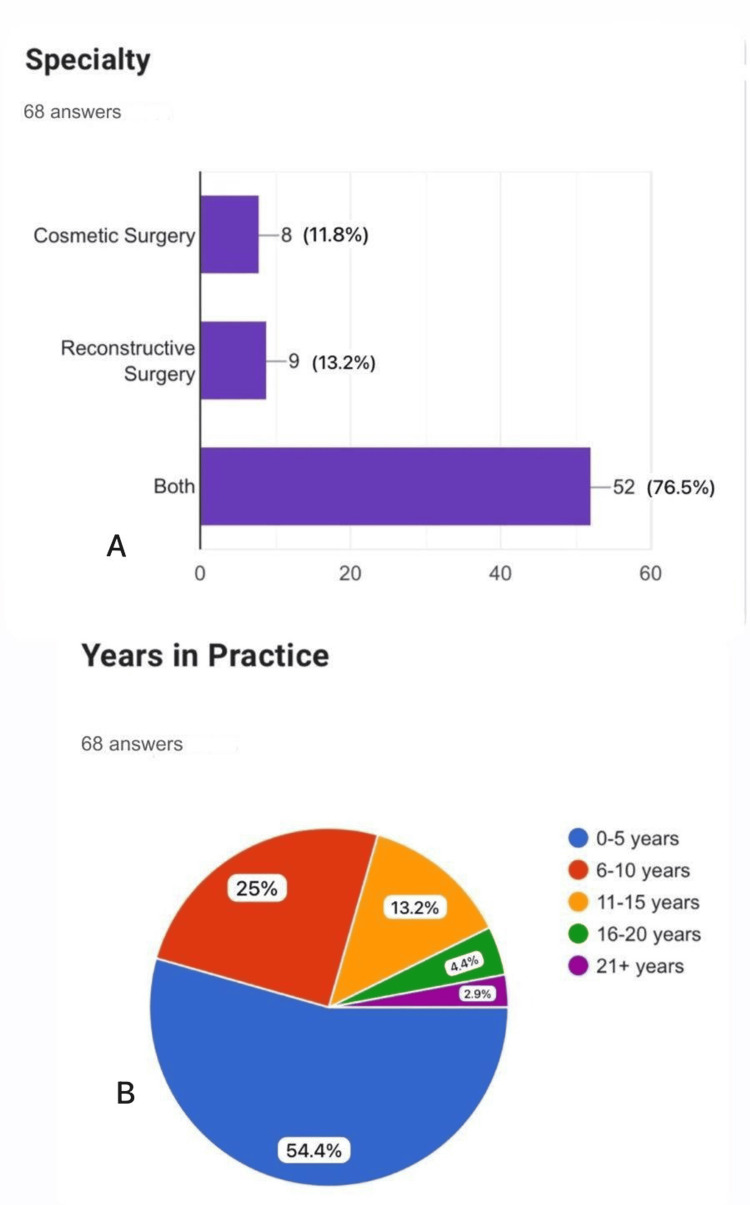
Speciality and years of practice of participants: (A) specialty of participants; (B) range of experience of participants

The survey respondents represented a diverse cross-section of the plastic surgery community. The majority of participants were male (70.6%), with a significant portion practicing in North America (55.9%), followed by Europe (22.1%). Other regions represented included Asia (11.8%), Australia (5.9%), Africa (2.9%), and South America (1.5%) (Figure 1). The range of experience varied, with 54.4% of respondents having been in practice for 0-5 years, 25% for 6-10 years, 13.2% for 11-15 years, 4.4% for 16-20 years, and 2.9% for over 21 years. Regarding specialties, a large majority (76.5%) practiced both cosmetic and reconstructive surgery, while 11.8% focused solely on cosmetic surgery, and 13.2% on reconstructive surgery (Figure 2).

Influence of social media on patient expectations

**Figure 3:**
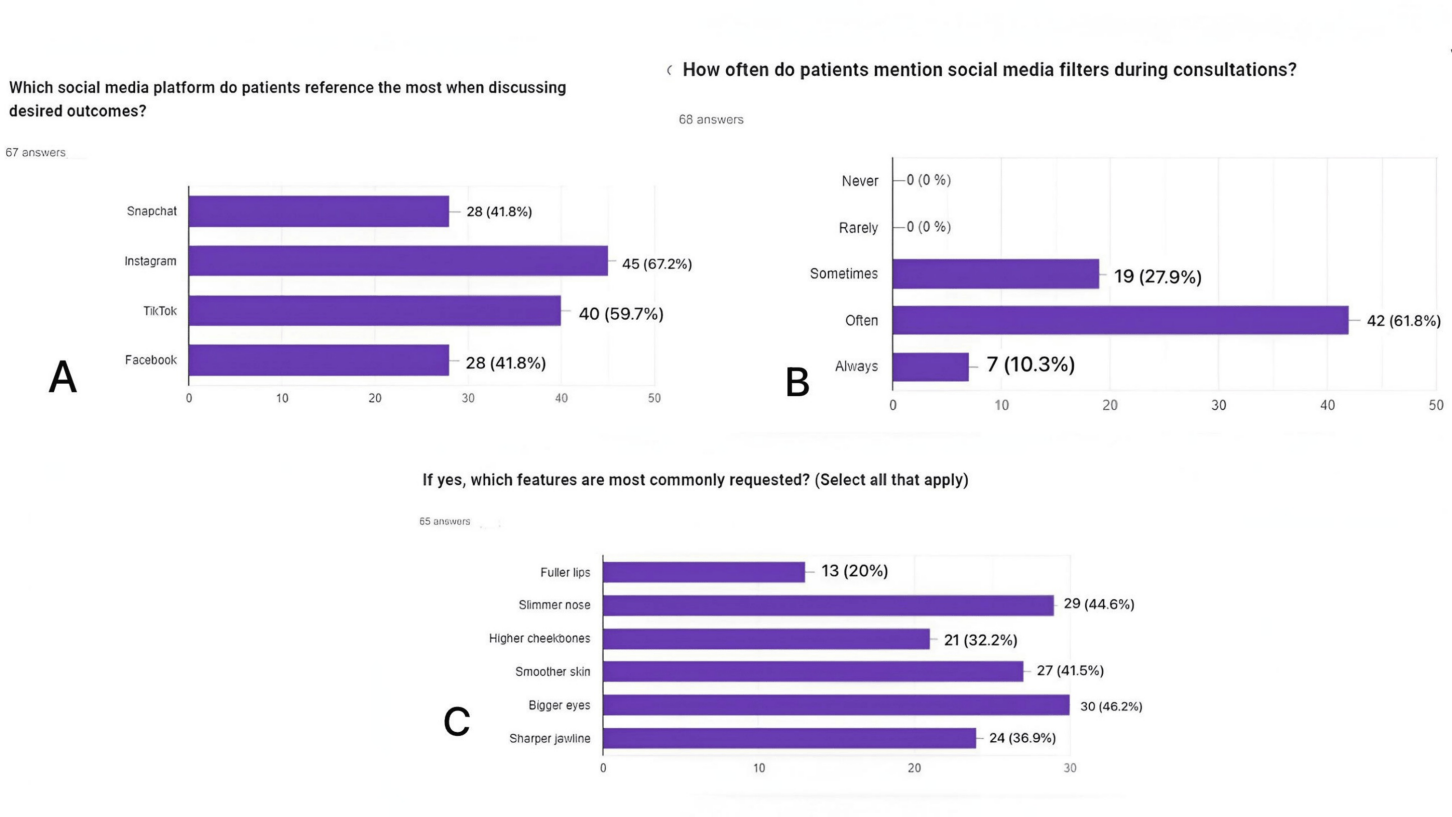
Social media platform used by patients,social media filters used and types of features most requested. (A) Which social media platform do patients reference the most when discussing desired outcomes? (B) How often do patients mention social media filters during consultations? (C) If yes, which features are most commonly requested?

None of the respondents reported that patients never or rarely mention filters; instead, 27.9% said it happens sometimes, 61.8% often, and 10.3% always. This suggests that social media filters have become a pervasive influence in the consultation room (Figure 3B).

Instagram was the most frequently cited platform, with 67.2% of respondents indicating it was mentioned by patients, followed closely by TikTok (59.7%), Snapchat (41.8%) and Facebook (41.8%). The types of features patients most commonly requested, influenced by these platforms, included bigger eyes (46.2%), a slimmer nose (44.6%), smoother skin (41.5%), and a sharper jawline (36.9%) (Figure 3).

Ethical considerations and psychological impact

A majority of respondents (57.4%) indicated they had some ethical concerns, with (n= 30.9%) expressing major concerns. Only a small minority (2.9%) reported few ethical concerns, and none indicated having no concerns at all.

In terms of psychological impacts, 86.6% of respondents reported observing negative effects on patients due to their reliance on social media filters. The most commonly observed psychological conditions included body dysmorphic disorder (51.6%), depression (48.4%), increased anxiety (38.7%), and lower self-esteem (35.5%). The issue of unrealistic body image expectations was also significant, with 32.3% of respondents noting this concern.

These findings highlight the dual challenge faced by plastic surgeons: managing the clinical aspects of surgery while also addressing the psychological well-being of their patients. The prevalence of psychological impacts indicates that the desire to achieve a “filtered” appearance can have far-reaching consequences beyond physical changes.

Professional practices and adaptations

The most common strategies included showing before-and-after photos (69.1%), explaining the limitations of surgery (67.6%), and encouraging realistic expectations (41.2%). Additionally, 26.5% of respondents used digital morphing tools to help patients visualize potential outcomes. However, the use of these tools was approached cautiously, as they can sometimes exacerbate unrealistic expectations if not used appropriately.

Interestingly, while 39.7% of respondents reported using social media filters themselves to demonstrate potential outcomes to patients, a larger proportion 60.3%) did not use these tools. Approximately 40% of respondents reported that social media filters had significantly changed their approach, while 45.6% noted moderate changes. Only a small fraction (n= 14.7%) reported slight changes, and none indicated that their approach had not changed at all.

Patient outcomes

Regarding patient outcomes, the survey revealed that 74.6% of respondents had refused a patient’s request for a procedure due to unrealistic expectations influenced by social media filters. Among those who had refused such requests, 49% reported doing so rarely, 43.1% sometimes, and 7.8% often.

In terms of patient satisfaction, n= 47.1% of respondents tracked the satisfaction rates of patients influenced by social media filters. Among those who had done so, n= 45.7% reported that these patients had higher satisfaction rates compared to those not influenced by social media, while n= 31.4% reported similar satisfaction levels. Interestingly, none reported lower satisfaction rates, suggesting that while these patients may have higher expectations, they are not necessarily less satisfied with the outcomes.

Around 79.4% of respondents noted the trend of higher incidence of revision surgeries among patients influenced by social media. The most common reasons for revision surgeries included unrealistic expectations (n= 67.3%), dissatisfaction with results (n= 34.5%), and influence from new social media trends (n= 38.2%). These findings suggest that while initial satisfaction may be achieved, the evolving nature of social media trends can lead to ongoing dissatisfaction and the desire for further alterations.

Regulatory considerations and professional development

Over half of the respondents (51.5%) strongly agreed that legal regulations should be implemented, while 38.2% agreed, and 10.3% were neutral. None of the respondents disagreed with this statement, reflecting a consensus on the need for greater oversight.

In terms of professional development, a significant majority (77.9%) supported the need for specialized training for plastic surgeons to handle consultations influenced by social media. Additionally, 63.2% of respondents had attended workshops or training sessions focused on the impact of social media on plastic surgery, and 85.1% expressed interest in attending such sessions in the future. 

## Discussion

The influence of social media on plastic surgery has emerged as a significant force, reshaping patient expectations, surgeon-patient interactions, and the ethical landscape of the field. This discussion will analyze the findings from our survey in light of existing literature, exploring the implications for clinical practice, ethical considerations, and future trends in plastic surgery.

Social media filters and changing patient expectations

The pervasive influence of social media filters on patient expectations is one of the most notable trends identified in our survey. The majority of surgeons reported that patients often reference social media filters during consultations, with platforms like Instagram, TikTok, and Snapchat being the most frequently mentioned. This aligns with existing literature, which has documented the rise of "Snapchat dysmorphia," a term coined to describe the phenomenon where patients seek to emulate the idealized, often unrealistic, appearance created by filters [13]. The impact of these filters on patient expectations cannot be understated. They not only set a standard of beauty that is virtually unattainable but also blur the line between reality and fantasy, leading patients to desire physical changes that may not be feasible through surgery alone.

Our survey results indicate that the features most commonly requested by patients--such as bigger eyes, a slimmer nose, and a sharper jawline--reflect the aesthetic ideals popularized by social media filters. This is consistent with findings from other studies, which have shown that social media platforms often promote a narrow and homogeneous standard of beauty, characterized by features like high cheekbones, full lips, and smooth, poreless skin [14]. These features are often the result of digital manipulation rather than natural variation, which can create unrealistic expectations for patients seeking to achieve similar results through plastic surgery.

The challenge for plastic surgeons lies in managing these expectations while maintaining ethical standards of practice. As our survey revealed, many surgeons have adapted their consultation practices to address the influence of social media, using tools like before-and-after photos and digital morphing software to help patients visualize realistic outcomes. However, the use of digital tools in consultations must be approached with caution. While they can be effective in managing expectations, they also carry the risk of perpetuating the very issues they aim to mitigate--namely, the pursuit of an idealized appearance that may not be achievable in real life [15].

Ethical considerations in the age of social media

The ethical implications of social media filters in the context of plastic surgery are profound and multifaceted. Our survey results indicate that a significant number of surgeons harbor ethical concerns about the influence of these filters on patient requests. These concerns are well-founded, as the desire to replicate a filtered appearance can lead to unrealistic expectations, disappointment with surgical outcomes, and, in some cases, psychological harm.

The concept of "informed consent" is central to the ethical practice of plastic surgery. It requires that patients fully understand the risks, benefits, and limitations of a procedure before consenting to it. However, when patient expectations are shaped by social media filters, the informed consent process becomes more complex. Surgeons must not only explain the technical aspects of a procedure but also address the psychological motivations behind the patient's request, ensuring that they understand the difference between a digitally altered image and what can be realistically achieved through surgery [16].

The psychological impacts of social media filters are another critical ethical consideration. Our survey found that many surgeons have observed conditions such as body dysmorphic disorder (BDD), depression, and anxiety in patients who are heavily influenced by social media. These findings are supported by a growing body of literature linking social media use to mental health issues. For example, in a study published by Thai et al., the use of social media is associated with higher rates of anxiety, depression, and body dissatisfaction, particularly among young adults [17]. The desire to achieve a "filtered" appearance can exacerbate these issues, leading patients to pursue surgery as a means of correcting perceived flaws that may not exist in reality.

Given these ethical challenges, it is crucial for plastic surgeons to adopt a holistic approach to patient care, one that considers both the physical and psychological aspects of a patient's request. Surgeons should adopt a patient-centered consultation approach, actively listening to concerns while guiding patients toward realistic aesthetic goals. Utilizing preoperative imaging tools can help illustrate achievable results and highlight the limitations of digital enhancements. Additionally, educating patients about the artificial nature of social media filters through side-by-side comparisons of unedited vs. filtered images can help them develop more realistic expectations. Addressing psychological concerns with nonjudgmental language and normalizing diverse beauty standards can further support patients in making informed decisions. When necessary, referral to psychological counseling should be presented as a proactive step toward mental well-being rather than a barrier to surgery. This may involve collaborating with mental health professionals to assess patients who exhibit signs of BDD or other psychological conditions before proceeding with surgery. In some cases, it may be necessary to refuse a patient's request if their expectations are deemed unrealistic or if surgery is likely to exacerbate underlying mental health issues [18].

The role of social media in shaping beauty standards

The role of social media in shaping contemporary beauty standards cannot be overstated. Platforms like Instagram and TikTok have become the primary venues for the dissemination of beauty ideals, often driven by influencers and celebrities who use filters to enhance their appearance. This phenomenon has been described as the "Instagram effect," where the curated, idealized images presented on social media set unattainable standards of beauty for the average person [19].

Our survey results highlight the significant impact of these standards on patient behavior, with many seeking to emulate the looks popularized by social media influencers. This trend has been observed in other studies as well. For instance, a study published by Sultan et al., found that the majority of patients seeking facial cosmetic surgery were motivated by the desire to achieve a more "Instagrammable" appearance, often influenced by the images they saw on social media [20, 21].

The homogenization of beauty standards is another concerning aspect of social media's influence. As filters and digital manipulation become more widespread, there is a growing tendency for individuals to aspire to a narrow set of aesthetic ideals, often characterized by features that are not representative of natural human diversity. This can lead to a loss of individuality and a decrease in self-acceptance, as people feel pressured to conform to these ideals [19,21].

The implications of this trend for plastic surgery are significant. Surgeons must navigate the fine line between helping patients achieve their aesthetic goals and reinforcing unrealistic beauty standards. This requires a deep understanding of the cultural context in which these requests are made, as well as a commitment to promoting diversity and individuality in their practice. Surgeons should strive to educate patients about the dangers of homogenized beauty ideals and encourage them to embrace their unique features rather than conforming to an unattainable standard [22].

Psychological impact of social media filters

The psychological impacts of social media filters are a major concern for both plastic surgeons and mental health professionals. Our survey results indicate that the majority of surgeons have observed negative psychological effects in patients influenced by social media, including BDD, depression, anxiety, and low self-esteem. These findings are consistent with a growing body of research that links social media use to mental health issues, particularly among young people.

Body dysmorphic disorder is a particularly concerning condition that has been linked to social media use. BDD is characterized by an obsessive preoccupation with perceived flaws in one's appearance, which are often minor or nonexistent. Individuals with BDD may spend excessive amounts of time scrutinizing their appearance in mirrors, comparing themselves to others, or seeking reassurance from others about their looks [23]. Social media platforms, with their constant stream of idealized images, can exacerbate these behaviors by reinforcing the idea that one's appearance is flawed or inadequate.

The prevalence of BDD among patients seeking cosmetic surgery is notably high. A study published in Cyberpsychology, Behavior, and Social Networking by Griffiths et al., found that approximately 15% of patients seeking cosmetic procedures meet the diagnostic criteria for BDD [24]. This is significantly higher than the prevalence of BDD in the general population, which is estimated to be around 2-3% [25]. The desire to achieve a "filtered" appearance can be particularly problematic for individuals with BDD, as it may lead to repeated surgeries in an attempt to correct perceived flaws that are not based on reality.

Depression and anxiety are other common psychological issues associated with social media use. The constant comparison to others, the pressure to present a perfect image online, and the fear of missing out (FOMO) can all contribute to feelings of inadequacy and low self-worth. This is particularly true for young adults, who are the most frequent users of social media and are also in a critical stage of identity formation [26]. For these individuals, the desire to undergo plastic surgery may be driven more by a need to fit in or gain social approval than by a genuine desire for self-improvement.

Given the psychological risks associated with social media use, it is essential for plastic surgeons to screen patients for mental health issues before proceeding with surgery. This may involve using standardized screening tools, such as the Body Dysmorphic Disorder Questionnaire (BDDQ), to assess patients for signs of BDD or other conditions. In cases where psychological issues are identified, referral to a mental health professional should be considered as part of the pre-surgical assessment process [27].

Professional practices and adaptations

Our survey results indicate that plastic surgeons are increasingly adapting their practices to address the challenges posed by social media filters. The most common strategies reported by respondents included using before-and-after photos, explaining the limitations of surgery, and encouraging realistic expectations. These practices are essential for managing patient expectations and ensuring that they have a clear understanding of what can and cannot be achieved through surgery.

The use of digital morphing tools is another adaptation that has gained popularity among plastic surgeons. These tools allow surgeons to create a digital representation of what the patient might look like after surgery, helping to bridge the gap between patient expectations and realistic outcomes. However, as our survey results suggest, the use of these tools must be approached with caution. While they can be effective in managing expectations, they also carry the risk of perpetuating unrealistic standards of beauty if not used judiciously [28].

In addition to these practical adaptations, many surgeons are also engaging in ongoing professional development to better understand the impact of social media on their practice. This includes attending workshops and training sessions focused on the psychological and ethical implications of social media filters, as well as collaborating with mental health professionals to provide comprehensive care to their patients. The strong support among survey respondents for more regulations on social media platforms further highlights the need for a collective response to the challenges posed by social media in the field of plastic surgery.

Future directions and recommendations

The influence of social media on plastic surgery is likely to continue growing as these platforms become more integrated into daily life. As this trend evolves, it will be important for the plastic surgery community to take proactive steps to address the challenges it presents. Based on our survey findings and the existing literature, several recommendations can be made for the future of plastic surgery in the age of social media.

Enhanced Patient Education

Surgeons should place a greater emphasis on educating patients about the differences between digitally altered images and real-life outcomes. This could involve using visual aids, such as unfiltered before-and-after photos, to provide a more accurate representation of what can be achieved through surgery.

Psychological Screening

Given the high prevalence of mental health issues among patients influenced by social media [29], it is essential to incorporate psychological screening into the pre-surgical assessment process. This could include the use of standardized screening tools, as well as collaboration with mental health professionals to ensure that patients are receiving appropriate care.

Ethical Guidelines

The plastic surgery community should develop and promote ethical guidelines for managing consultations influenced by social media. These guidelines could address issues such as the use of digital morphing tools, the management of unrealistic expectations, and the ethical considerations of performing surgery on patients with psychological vulnerabilities.

Advocacy for Regulation

Plastic surgeons should advocate for increased regulation of social media platforms to address the impact of filters on body image. This could involve working with policymakers to develop guidelines for the use of filters in advertising and promoting transparency in the portrayal of cosmetic procedures on social media.

Professional Development

Ongoing education and training will be essential for plastic surgeons to stay informed about the latest trends and challenges related to social media. This could include attending workshops, participating in professional networks, and engaging in research on the impact of social media on patient behavior.

Promoting Diversity

Finally, plastic surgeons should take an active role in promoting diversity and individuality in their practice. This could involve encouraging patients to embrace their unique features and challenging the narrow beauty ideals often perpetuated by social media.

Limitations of the study

Sample Size

The study is based on a relatively small sample size of 68 plastic surgeons, which may not fully represent the diversity of practices, regions, or experiences within the global plastic surgery community. A larger sample size would provide more robust and generalizable data. 

Geographical Limitations

While the survey included responses from different regions, the majority of participants were based in the United States and Europe. This geographic concentration may limit the applicability of the findings to other regions where cultural attitudes toward beauty and social media usage may differ significantly.

Survey Design and Bias

The use of self-reported data through an online survey can introduce response bias. Surgeons may underreport or overemphasize certain aspects of their experiences due to personal biases or perceptions of social desirability. The anonymous nature of the survey may help reduce bias, but it cannot be fully eliminated.

Lack of Patient Perspectives

The study focuses solely on the perspectives of surgeons, without including data from patients. Incorporating patient viewpoints would provide a more comprehensive understanding of how social media filters influence the decision-making process and patient satisfaction in plastic surgery.

Psychological Assessment Limitations

While the study touches on the psychological impacts observed by surgeons, it lacks detailed psychological assessments of patients. Relying on surgeon observations to diagnose conditions like Body Dysmorphic Disorder (BDD) or anxiety can be imprecise. Collaboration with mental health professionals or incorporating validated psychological screening tools could enhance the depth of the findings.

Temporal Scope

The survey was conducted over a limited time frame (June to August 2024), which may not capture long-term trends in how social media filters influence plastic surgery demands. As social media trends evolve quickly, a longitudinal study could offer more insight into changing patient expectations over time.

Platform-Specific Findings

Although the study identifies Instagram, TikTok, and Snapchat as the most frequently cited platforms, it does not explore other emerging platforms or consider how different social media algorithms may uniquely affect patient expectations. Additionally, it focuses mainly on facial features, potentially overlooking the influence of social media filters on body contouring procedures.

Technological Variations

The study mentions the use of digital morphing tools by some surgeons but does not account for the variability in how these tools are implemented. Differences in the quality and accuracy of these tools could impact patient expectations and outcomes, and this variation is not fully addressed.

Ethical and Regulatory Generalizations

The study touches on the ethical concerns and the call for greater regulation but lacks specific details on how different regions or countries currently regulate social media's influence on plastic surgery. It also does not delve into how differing legal frameworks might affect surgeons' practices in various jurisdictions.

Focus on Cosmetic Surgery

Although the study includes both cosmetic and reconstructive surgeons, the emphasis on aesthetic procedures influenced by social media filters may overshadow the broader ethical concerns faced by reconstructive surgeons. Future studies could expand on the impact of social media in the reconstructive domain, where patient needs and motivations differ significantly.

## Conclusions

The influence of social media filters on plastic surgery is a complex and multifaceted issue that requires careful consideration by plastic surgeons, mental health professionals, and policymakers alike. Our survey results underscore the significant impact of these filters on patient expectations, psychological well-being, and ethical practice in the field of plastic surgery. As social media continues to evolve, it will be essential for the plastic surgery community to adapt to these changes thoughtfully, prioritizing patient well-being and ethical integrity in their practice. By taking a proactive approach to education, psychological screening, ethical guidelines, and advocacy, plastic surgeons can help mitigate the negative effects of social media on body image and promote a more realistic and inclusive standard of beauty.

## Appendices

Table 1 shows the questionnaire distributed to the respondents.

**Table 1:** The Impact of Social Media Filters on Plastic Surgery

The Impact of Social Media Filters on Plastic Surgery
Age
Gender
Location
Professional Information:
Years in Practice
Specialty
Impact of Social Media Filters
How often do patients mention social media filters during consultations?
Which social media platform do patients reference the most when discussing desired outcomes?
Have you noticed an increase in requests for specific features due to social media filters (e.g., fuller lips, slimmer nose)?
If yes, which features are most commonly requested? (Select all that apply)
How do you feel social media filters impact patient expectations?
Do you believe social media filters have increased the overall demand for plastic surgery in your practice?
How do you handle consultations when patients reference social media filters?
Do you use social media filters to demonstrate potential outcomes to patients?
Professional Observations
How has the trend of social media filters changed the way you approach patient consultations?
What is your perspective on the ethical considerations of social media filters influencing plastic surgery requests?
Would you support more regulations on social media platforms to address the impact of filters on body image?
Patient Demands and Trends
How often do patients bring reference photos altered by social media filters to their consultations?
Do you think social media filters contribute to a specific demographic (age/gender) requesting plastic surgery more frequently?
If yes, which demographic group is most influenced?
Have you observed any psychological impacts on patients due to their reliance on social media filters?
If yes, what psychological impacts have you noticed? (Select all that apply)
Do you believe there is a correlation between the use of social media filters and the rise in minimally invasive procedures (e.g., fillers, Botox)?
Which minimally invasive procedures are most requested due to social media filters? (Select all that apply)
Professional Practices and Adaptations
How often do you use digital imaging tools to help patients visualize potential outcomes?
What is your primary goal during consultations influenced by social media filter requests?
Do you believe that social media platforms should provide disclaimers about the use of filters and their potential impact on body image?
Have you ever refused a patient's request for a procedure due to unrealistic expectations influenced by social media filters?
If yes, how often do you refuse such requests?
Education and Training
Do you think there is a need for specialized training for plastic surgeons on handling social media-influenced consultations?
Have you attended any workshops or training sessions focused on the impact of social media on plastic surgery?
Would you be interested in attending such workshops or training sessions in the future?
Technological and Ethical Considerations
Do you use social media to promote your practice?
If yes, which platforms do you use? (Select all that apply)
Do you believe the portrayal of plastic surgery results on social media is generally accurate or misleading?
How do you ensure the ethical promotion of your plastic surgery services on social media? (Select all that apply)
What changes do you anticipate in the demand for plastic surgery due to the ongoing influence of social media filters?
What measures do you think should be taken by the plastic surgery community to address the impact of social media filters? (Select all that apply)
Patient Communication and Counseling
How do you address patient concerns about looking "unnatural" after surgery due to social media influences?
Do you find it necessary to discuss the psychological effects of social media filters with patients during consultations?
What resources do you provide to patients who express concerns influenced by social media? (Select all that apply)
Trends and Observations
In your experience, have you seen an increase in younger patients requesting plastic surgery due to social media?
Which procedures have you noticed a significant increase in demand for due to social media trends? (Select all that apply)
Professional Development
Do you collaborate with other professionals (e.g., dermatologists, mental health professionals) to address issues arising from social media influences?
If yes, how often do you collaborate with these professionals?
What additional support or resources would help you manage the impact of social media on your practice? (Select all that apply)
Patient Outcomes
Have you tracked the satisfaction rates of patients who were influenced by social media filters in their decision-making?
If yes, how do the satisfaction rates of these patients compare to those who were not influenced by social media?
Do you find that patients influenced by social media filters have a higher incidence of revision surgeries?
If yes, what is the most common reason for revision surgeries in these patients?
Ethical and legal considerations
Do you think there should be legal regulations governing the use of social media filters in advertising for plastic surgery?
